# Chronic mineral oil administration increases hepatic inflammation in wild type mice compared to lipocalin 2 null mice

**DOI:** 10.1038/s41374-021-00672-9

**Published:** 2021-09-13

**Authors:** Erawan Borkham-Kamphorst, Ute Haas, Manuela Pinoé-Schmidt, Ali T. Abdallah, Ralf Weiskirchen

**Affiliations:** 1 Institute of Molecular Pathobiochemistry, Experimental Gene Therapy and Clinical Chemistry, RWTH Aachen University Hospital, Aachen, Germany; 2 Interdisciplinary Center for Clinical Research, University Hospital RWTH, Aachen, Germany

**Keywords:** Molecular biology, Diseases

## Abstract

Lipocalin 2 (LCN2), an acute-phase protein produced during acute liver injury, plays an important role in the innate immune response against bacterial infection via iron scavenging. LCN2 further influences neutrophil development and physiology leading to increased inflammatory responses. We investigated the roles of LCN2 in chronic inflammation and fibrosis, using repeated carbon tetrachloride (CCl_4_) in mineral-oil injection. Surprisingly, mice treated with the mineral oil vehicle alone showed liver inflammation, evidenced by neutrophil and monocyte-macrophage infiltration. Fluorescence-activated cell sorting (FACS) of isolated liver leukocytes showed significantly high CD45^+^ leukocyte concentrations in CCl_4_ mice, but no difference of Ly6G^+^ neutrophils between mineral oil and CCl_4_ application. Liver CD11b^+^ F4/80^+^ cells counted higher in CCl_4_ mice, but the proportions of Gr1^high^, an indicator of inflammation, were significantly higher in mineral oil groups. Liver myeloperoxidase (MPO), expressed in neutrophils and monocytes, showed higher levels in wild type mice compared to *Lcn2*^−/−^ in both mineral-oil and CCl_4_ treated groups. Hepatic and serum LCN2 levels were remarkably higher in the mineral oil-injected wild type group compared to the CCl_4_. Wild type animals receiving mineral oil showed significantly higher inflammatory cytokine- and chemokine mRNA levels compared to *Lcn2*^−/−^ mice, with no differences in the CCl_4_ treated groups. RNA sequencing (RNA-Seq) confirmed significant downregulation of gene sets involved in myeloid cell activation and immune responses in *Lcn2* null mice receiving chronic mineral oil versus wild−type. We observed significant upregulation of gene sets and proteins involved in cell cycle DNA replication, with downregulation of collagen-containing extracellular matrix genes in *Lcn2*^–/–^ mice receiving CCl_4,_ compared to the wild type. Consequently, the wild type mice developed slightly more liver fibrosis compared to *Lcn2*^−/−^ mice, evidenced by higher levels of collagen type I in the CCl_4_ groups and no liver fibrosis in mineral oil-treated mice. Our findings indicate that serum and hepatic LCN2 levels correlate with hepatic inflammation rather than fibrosis.

## Abstract (web summary)

Chronic mineral-oil intraperitoneal administration induces hepatic inflammation through innate immune responses via myeloid cell activation. *Lcn2* null mice develop less inflammation due to defective genes involved in myeloid cell activation, and downregulated gene sets for collagen-containing extracellular matrix, leading to mitigation of the liver fibrosis. *Lcn2* null mice show enriched expression of genes responsible for DNA damage and cell cycle DNA replication compared to wild-type upon chronic CCl_4_ liver injury.

## Introduction

Lipocalin 2 (LCN2) or neutrophil gelatinase-associated lipocalin (NGAL) in human is a 24 kDa secretory glycoprotein, isolated from specific granules of neutrophils^1^. LCN2 plays an important role in innate immune responses against bacterial infections by sequestering iron-containing siderophores^2,3^. Consequently, *Lcn2* knockout (*Lcn2*^*−/−*^) mice are susceptible to infections, especially to siderophore-producing bacteria^4^. In *Lcn2*^−/−^ mice neutrophil maturation and functionality are affected, which is reflected by increased band cells in circulation and higher amounts of neutrophil count and reduced migratory activity, adhesion, tissue extravasation, phagocytosis, and chemotaxis^5^. *Lcn2*^−/−^ mice, therefore, are sensitive to pathogens, in part due to impaired functioning in neutrophils^5–7^. LCN2 induces the production of cytokines and chemokines to promote the migration and phagocytosis of macrophages, resulting in stronger inflammatory responses in wild type compared to *Lcn2*^−/−^ mice^6,7^. However, in non-pathogen-driven inflammatory conditions such as non-alcoholic steatohepatitis (NASH) and alcoholic steatohepatitis (ASH), LCN2 plays detrimental roles in the liver through neutrophil infiltration^8–10^. On the contrary, LCN2 showed protective effects in bacterial infections and animal models of liver injury^11–15^. Furthermore, human serum LCN2 is a biomarker of acute-on-chronic liver failure and prognosis in cirrhosis^16^ and reported to correlate to non-alcoholic fatty liver disease (NAFLD)^17^, ASH disease severity, and the model for end-stage liver disease (MELD) score^18^. We and others did not find systemic LCN2 levels to directly correlate to impaired liver function and disease severity^15,19^. We here used the 8 week-repeated carbon tetrachloride (CCl_4_) intraperitoneal injections of female C57BL/6 wild type and *Lcn2*^*−/−*^ mice to evaluate the role of LCN2 in hepatic inflammation and fibrosis. Unexpectedly, mice that received the mineral oil vehicle showed liver inflammation as evidenced by neutrophil and monocyte-macrophage infiltration. Fluorescence-activated cell sorting (FACS) of isolated liver leukocytes revealed significantly higher quantities of liver CD45^+^ leukocytes in CCl_4_ mice, with no marked differences of Ly6G^+^ neutrophils in the mineral oil- and CCl_4_-treated groups. Levels of liver CD11b^+^ F4/80 cells were higher in CCl_4_ mice, but the proportions of the inflammation indicator Gr1^high^, were significantly higher in the mineral oil groups. Hepatic expression of myeloperoxidase (MPO), the pro-inflammatory enzyme expressed in neutrophils and monocytes, was found to be higher in wild type mice as compared to *Lcn2*^−/−^ mice after both mineral oil and CCl_4_ administration. The levels of hepatic and serum LCN2 were significantly higher in the mineral oil-injected wild type group relative to CCl_4_. Wild type animals receiving mineral oil showed significantly higher levels of inflammatory cytokine- and chemokine mRNA compared to *Lcn2*^−/−^ mice, while the expression in the CCl_4_ groups was similar. RNA sequencing (RNA-Seq) showed significant downregulation of gene sets involved in myeloid cell activation and immune responses in *Lcn2* null mice treated with mineral oil versus wild type. Surprisingly, we noted significant upregulation of gene sets and proteins involved in DNA replication and cell cycle and downregulation of collagen-containing extracellular matrix genes in *Lcn2*^−/−^ mice receiving CCl_4_, compared to the wild type. Consequently, CCl_4_ administered wild type mice developed slightly more liver fibrosis than *Lcn2*^−/−^ mice, evidenced by higher levels of collagen type I in the CCl_4_ groups. No significant liver fibrosis was observed in mice treated with mineral oil.

## Materials and methods

### Animal experiments and specimen collections

Eight-week-old female C57BL/6 wild type and *Lcn2*^−/−^ mice^4^ were subjected to intraperitoneal (i.p.) injection of 0.8 ml/kg body weight CCl_4_ diluted in mineral oil twice weekly for 8 weeks. The mineral oil vehicle was applied to control animals. Mice were sacrificed 48 hours upon the last injection, heart blood samples were collected followed by liver perfusion. Liver specimens were snap-frozen in liquid nitrogen and stored at −80 °C for protein and RNA isolation. Tissue frozen sections and paraffin-embedded sections were prepared for histology. All animal protocols complied with the guidelines for animal care approved by the German Animal Care Committee (Landesamt für Naturschutz, Umwelt und Verbraucherschutz Nordrhein–Westfalen (LANUV) located in Recklinghausen, Germany; https://www.lanuv.nrw.de).

### Intrahepatic leukocyte isolations

Most of the liver tissue was used for intrahepatic leukocyte isolation and flow cytometric analysis as described^20^. In brief, livers were perfused with phosphate-buffered saline (PBS), minced with scissors, and subsequently digested for 30 min at 37 °C with collagenase type-IV (Worthington Biochemical, NJ, USA). To gain single-cell suspensions, the digested products were pressed through 70-μm cell strainers. A small aliquot was taken to stain with CD45 for assessment of the relative number of intrahepatic leukocytes (CD45^+^) to all liver cells. The remaining liver single-cell suspension was subjected to density gradient centrifugation in Lymphocyte Separation Medium (LSM-1077, PAA Laboratories, Cölbe, Germany) at 2000 rpm for 20 min at 25 °C. Upon centrifugation, the leukocytes were collected from the interphase. Cells were washed twice with Hank’s balanced salt solution containing 2% bovine serum albumin and 0.1 mM ethylenediaminetetraacetic acid (EDTA) and subjected to FACS.

### Flow cytometry

Six-color staining was performed using combinations of the following monoclonal antibodies: F4/80 (Serotec, Puchheim, Germany), CD115, CD4, CD11b (all from eBioscience, Frankfurt/Main, Germany), CD45, Gr1/Ly6C, Ly6G, NK, CD8, CD3, mouse immunoglobulin (Ig) G1, rat IgG2a, or hamster IgG isotype controls (all BD Bioscience, Heidelberg, Germany). Flow cytometric analysis was performed on a FACS-Canto (BD) and analyzed with FlowJo (Tree Star Inc., Ashland, OR, USA).

### RNA isolation, cDNA synthesis, and RT-qPCR

Liver tissue was homogenized in QIAzol lysis reagent (Qiagen, Hilden, Germany) in a Mixer Mill MM400 homogenizer (Retsch, Haan, Germany). Total RNA was isolated as described previously through phenol-chloroform extraction, isopropanol precipitation, and DNAse digestion followed by subsequent RNA clean up with PureLink RNA Mini kits (Invitrogen, Thermo Fisher Scientific, Darmstadt, Germany) according to manufacturer’s guidelines^21^. Primers for amplification were selected from the sequences deposited in the GenBank database using the online ProbeFinder Software (Universal Probe Library Assay Design Center, Roche, Mannheim, Germany). First-strand cDNA was synthesized from 1 µg RNA in 20 µl volume using SuperScript^TM^ II reverse transcriptase and random hexamer primers (Invitrogen). For quantitative real-time PCR (RT-qPCR), cDNA derived from 50 ng RNA (5 μl of 1:5 dilution of cDNA) was amplified in 25 µl volume using SYBR^®^ GreenER™ qPCR SuperMix for ABI PRISM^®^ (Invitrogen). The PCR conditions were set to 50 °C for 2 min, 95 °C for 10 min initial denaturation, followed by 40 cycles of 95 °C for 15 sec, and 60 °C for 1 min. Relative mRNA expression was normalized to the housekeeping gene glyceraldehyde 3-phosphate dehydrogenase (*GAPDH*) and calculated using 2^−ΔΔCT^ method^22^. All primers used in this study are given in Supplementary Table 1.

### RNA-Seq library preparation and sequencing

Quality checks of RNA samples were performed with the TapeStation 4200 using the Agilent RNA ScreenTape assay kit (Agilent, Waldbronn, Germany). Quantification was performed using the Quantus RNA System (Promega, Madison, WI, USA). According to the manufacturer’s instructions, cDNA libraries were produced employing the rapid MACE kit for 3′ end RNA Sequencing (GenXPro GmbH, Frankfurt am Main, Germany), using 100 ng input RNA. 20% (260 µl) of denatured and diluted PhiX was added to the final loading mixture to increase the mixture’s diversity and enhance the kit’s performance. The quality and quantity of the cDNA libraries were assessed using the 4200 TapeStation (D1000 screen tape assay) and the Quantus dsDNA system. All samples were sequenced on a NextSeq 500 sequencing system with the following run parameters: 76 cycles for read 1, 6 cycles for index 1. The cDNA libraries were generated using a TruSeq Stranded Total RNA Sample Preparation Kit (Illumina, San Diego, CA, USA).

### RNA-Seq primary analysis

Generation of the fastq files and removal of adapters were completed using the Illumina demultiplexing and conversion software bcl2fastq (https://support.illumina.com/sequencing/sequencing_software/bcl2fastq-conversion-software.html). Subsequently, RNA-Seq primary analysis was performed with the RNA-Seq pipeline of nf.core v3.0^23^ using standard parameters. For instance, quality control of the sequenced transcripts was done with online routine fastqc v0.11.9 (http://www.bioinformatics.babraham.ac.uk/projects/fastqc), library complexity was measured with preseq v2.0.3^24^. Adapter and quality trimming was performed using TrimGalore v.0.6.6 (https://www.bioinformatics.babraham.ac.uk/projects/trim_galore/). Subsequently, the reads were aligned to the mm10 reference genome sequence using STAR v2.6.1d^25^ with default parameters. In the quantification step, reads were counted with the quasi-mapping quantification tool Salmon^26^.

### Differential expression analysis

Differential expression analysis (DEA) was then performed in R using the DESeq function of the DESeq2 v1.30.0 package^27^, with local fit type (fitType = “local”), without LFC modulation (betaPrior = False) and using the Wald test as significance test. Genes with log fold change >0.56 (FC ≥ 1.5) and corrected *p* value ≤0.05 were considered as differentially expressed.

### GO enrichment analysis

GO enrichment analyses (GOA) for each of the comparisons were performed with the gprofiler2 package v0.2.0^28^, using the package’s gost function, making an ordered query (genes sorted by significance) with an adjusted *p*-value threshold of 0.05 and the multiple test correction method gSCS.

### Availability of data and code

For reproducibility, the script of the advanced analysis (DEA and GOA and various QC’s and all plots used in this manuscript) is accessible via GitHub (https://github.com/ATA82/HepaticInflammation_LipocalinNullMice). The data are accessible via GEO accession number GSE176093.

### Liver protein lysate preparation, SDS-PAGE, and Western blot analysis

Liver tissues were homogenized in a Mixer Mill MM400 homogenizer followed by ultrasound sonication in RIPA buffer containing 20 mM Tris-HCl (pH 7.2), 150 mM NaCl, 2% (w/v) NP-40, 0.1% (w/v) SDS, 0.5% (w/v) sodium deoxycholate, and the Complete™-mixture of proteinase inhibitors (Roche). Proteins were quantified using DC™ Protein assay kit I (Bio-Rad, Feldkirchen, Germany) according to the manufacturer’s instructions. Equal amounts of protein extracts (50–100 μg) were diluted with Nu-PAGE™ LDS electrophoresis sample buffer with 50 mM dithiothreitol (DTT) as a reducing agent. The samples were heated at 95 °C for 10 min and separated in 4–12% Bis-Tris gradient gels, using MES running buffers (all from Invitrogen). Proteins were electroblotted onto nitrocellulose membranes (Schleicher & Schuell BioScience GmbH, Dassel, Germany) and confirmed by Ponceau S staining. Non-specific binding sites were blocked with 5% (w/v) non-fat milk powder in Tris-buffered saline and 0.1% Tween 20 (TBST). All antibodies (Supplementary Table 2) were diluted in 2.5% (w/v) non-fat milk powder in TBST. Primary antibodies were detected using horseradish peroxidase (HRP)-conjugated anti-mouse-, anti-rabbit-, or anti-goat IgG (Invitrogen) and the SuperSignal chemiluminescent substrate (Pierce, Bonn, Germany).

### Immunohistochemical and immunofluorescence staining

Liver tissue sections were deparaffinized and rehydrated with xylene and decreasing graded ethanol, whereas antigen retrieval was performed by heating the sections in a 10 mM sodium citrate buffer (pH 6.0) in a steamer for 30 min. Tissue slides were blocked with avidin-biotin (DAKO, Hamburg, Germany), followed by incubation in a solution containing 2–5% animal serum of which the second antibody was made in 1% BSA and 0.05% Tween 20 in PBS (PBST). Primary antibodies were diluted in 1% BSA, 0.1% cold fish skin gelatin, 0.3% Triton X-100 in PBST to concentrations of 2–5 μg/ml and incubated at 4 °C overnight, while omitting the primary antibody was used for negative control. Nonspecific peroxidase blocking was performed with 3% H_2_O_2_ for 10 min. Sections were then incubated with biotinylated secondary antibodies (DAKO) for 1 h, followed by avidin-biotin conjugated peroxidase for 30 min. (VECTASTAIN® Elite ABC-HRP peroxidase kit (PK-6100), Vector Laboratories, Burlingame, CA, USA). The sections were developed using 3,3′-diaminobenzidine substrates (SIGMAFAST™, Sigma-Aldrich, Taufkirchen, Germany), counterstained with hematoxylin (DAKO), and mounted with DPX Mountant for histology (Sigma-Aldrich).

For Immunofluorescence staining, liver tissue sections were prepared as mentioned above. The sections were blocked with 5% serum originating from the animal that the 2nd antibody was made of in 1% BSA in PBST for 1 h at room temperature, followed by incubation of primary antibodies at 4 °C overnight. Subsequently, the Alexa Fluor® conjugated secondary antibodies (Invitrogen) were applied for 1 h at room temperature and the nuclei were counterstained with 4′,6-diamidino-2-phenylindole (DAPI). The tissue sections were mounted with coverslips using Permafluor mountant TA-030-FM (Thermo Fisher Scientific) and analyzed by fluorescence microscopy, using the Nikon Eclipse 80i microscope (Nikon, Tokyo, Japan), equipped with the NIS-Elements Vis software (version 3.22.01, Nikon, Amsterdam, The Netherlands).

### Statistics

For statistical analysis we used GraphPad Prism 6 (GraphPad Software, Inc., San Diego, CA). Statistical significance of differences between mean values was determined using one-way analysis of variance (ANOVA) followed by Tukey Honestly Significant Difference (Tukey HSD) for multiple comparison tests. Probability values given are *p  <  0.05, **p  <  0.01, and ***p  < 0.001, respectively.

## Results

### Long-term application of mineral oil and CCl_4_ induced hepatic LCN2 expression

**Fig. 1:**
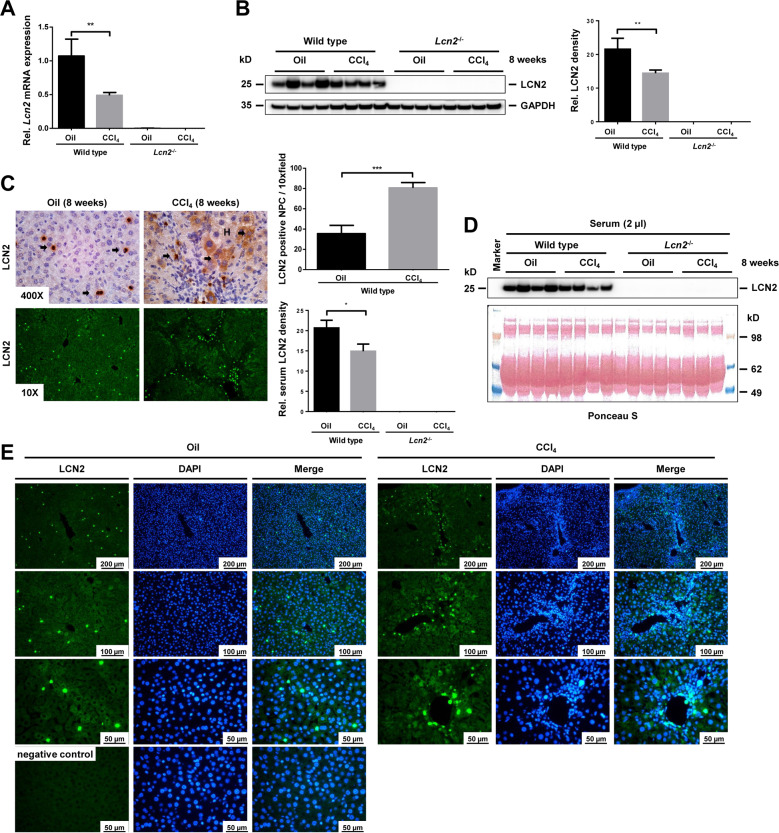
Long-term mineral oil and CCl_4_ induced liver LCN2 expression and increased serum LCN2. **A** Quantitative RT-PCR of *Lcn2* shows significantly higher levels of hepatic *Lcn2* in 8-week wild type oil administration compared to its CCl_4_ counterpart and no *Lcn2* expression in *Lcn2*^−/−^ mice. **B** Western blot confirmed significantly higher amounts of LCN2 protein in wild type mice receiving oil than CCl_4_. **C** Liver IHC of LCN2 reflects LCN2 positive NPC scattered homogeneously in mineral oil injection, while CCl_4_ livers showed positive in both NPC infiltrating cells and hepatocytes around central veins. The LCN2 positive NPC are significantly higher in the CCl_4_ liver. **D** Western blot analysis of serum (2 μl/lane) also showed that LCN2 correlates well with liver mRNA and protein levels with significantly higher levels in oil-injected animals. **E** Liver tissue immunofluorescence staining from wild type animals found LCN2 positive hepatocytes and NPC in the liver of oil- and CCl_4_-treated groups.

Upon eight week- mineral oil or CCl_4_ administration, induction of hepatic *Lcn2* mRNA and protein levels was significant in wild type livers (Fig. 1A, B). Immunohistochemical staining showed LCN2 positive non-parenchymal cells (NPC) scattered homogeneously within the tissue after mineral oil administration, while CCl_4_ livers showed positive LCN2 in both non-parenchymal infiltrating cells and hepatocytes around central veins (Fig. 1C). LCN2 positive NPC were double the amounts found in CCl_4_ compared to mineral oil injection (Fig. 1C). By contrast, serum LCN2 as assessed by Western blot analysis showed relatively higher oil injection in correlation to liver mRNA and protein levels (Fig. 1D). These findings indicate that besides NPC, the hepatic parenchymal cells such as hepatocytes produced and secreted LCN2 into circulation as shown in immunofluorescent staining of LCN2 positive hepatocytes in the oil and CCl_4_ groups (Fig. 1E).

### Repeated mineral oil injection-induced hepatic neutrophil infiltration

**Fig. 2:**
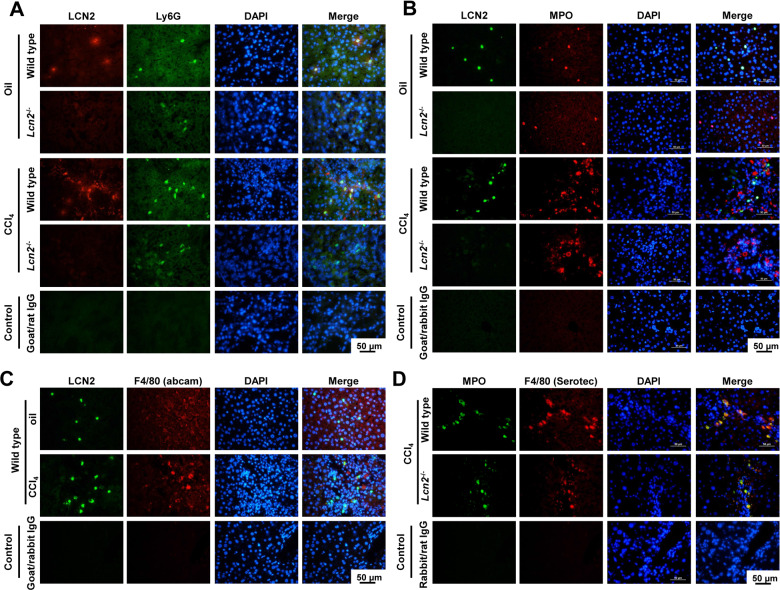
Immunofluorescence double staining of liver sections. **A** Frozen section double staining of LCN2 (*red*) and Ly6G (*green*) shows co-localization of LCN2 and Ly6G. **B** Paraffin section staining of LCN2 (*green*) and MPO (*red*) showed co-localization of LCN2 and MPO in wild type oil administration but co-localized only partially in CCl_4_ around central veins. **C** No co-localization of LCN2 (*green*) and F4/80 (*red*) in both oil and CCl_4_ administration. **D** CCl_4_ treated livers showed co-localization of MPO (*green*) and F4/80 *(red*) along the fibrotic central veins.

Repeated mineral oil i.p. injection-induced visible peritoneal granuloma (data not shown), while immunohistochemistry (IHC) and fluorescent staining showed LCN2 expression in liver NPC. We performed double staining of (i) LCN2 with lymphocyte antigen 6 complex locus G6D (Ly6G), expressed in myeloid-derived cells such as monocytes, granulocytes, and neutrophils, (ii) LCN2 with myeloperoxidase (MPO) that is abundant in neutrophils, and (iii) LCN2 with F4/80, a marker of Kupffer cells, monocytes, and macrophages. LCN2 double staining showed to co-localize with Ly6G and MPO in oil-injected livers, but only partially co-localization in CCl_4_ groups, while no co-localization of LCN2 with F4/80 (Fig. 2A–C) was found, indicating that chronic injection of both oil and CCl_4_ induced neutrophil infiltration and activated LCN2 production. Interestingly, significant co-localization of MPO and F4/80 along fibrotic central areas in the liver of animals that received CCl_4_ were noticed (Fig. 2D), possibly indicating that engulfed macrophages contained MPO.

### *Lcn2* null decreased neutrophil infiltration after both mineral oil and CCl_4_ administration as compared to the wild type mice

**Fig. 3:**
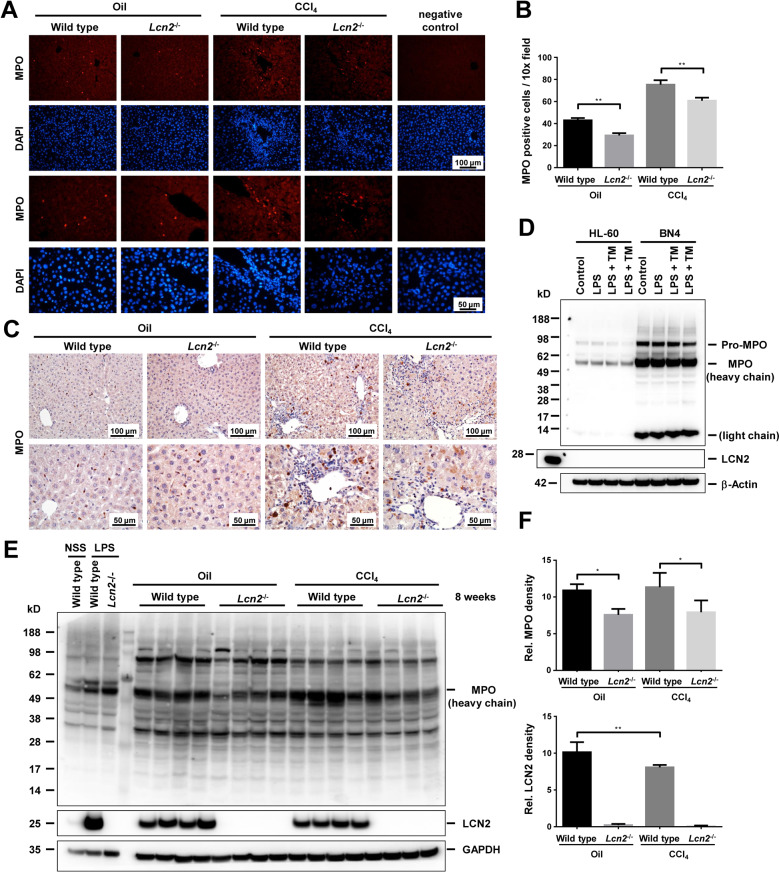
*Lcn2* null mice have decreased MPO^+^ cells and MPO protein after mineral oil and CCl_4_ administration. **A** IF staining shows lower numbers of MPO^+^ cells in *Lcn2*^−/−^ receiving oil and CCl_4_ compared to the wild type. **B** The average cell counts of MPO^+^ cells/10 x fields (10 fields/animal) were confirmed significantly lower in *Lcn2*^−/−^ livers. **C** IHC staining of MPO^+^ cells corresponds with immunofluorescence. **D** Western blot of HL-60 and NB4 cell lines incubated with LPS and tunicamycin (TM) were used as positive controls of the MPO antibody. The promyelocytic cell lines derived from human leukemia do not produce LCN2 upon LPS or LPS and TM application, HepG2 stimulated with IL-1β used as a positive control for LCN2 production and β-actin as a loading control. **E** Western blot of liver lysates showed higher amounts of MPO protein in wild type animals treated with oil and CCl_4_ compared to *Lcn2*^−/−^. Mice injected with LPS for 6 h were used as positive controls. Mineral oil and CCl_4_ induced LCN2 production with GAPDH as loading controls. **F** MPO and LCN2 Western blots were quantified and confirmed significantly lower MPO levels in *Lcn2*^*−/−*^, while the wild type mice receiving oil showed higher amounts of LCN2 compared to the CCl_4_ group.

Several publications state LCN2 to promote liver injury and inflammation in alcoholic steatohepatitis and nonalcoholic steatohepatitis (NASH)^8–10^ and LCN2 deficiency to reduce the migration of neutrophils^5^. We stained MPO, the specific marker of neutrophils, and found wild type livers to contain significantly more MPO-positive cells in both oil and CCl_4_ groups (Fig. 3A–C). MPO expression is restricted to myeloid cells and is synthesized and stored in azurophilic (primary) granules of neutrophils during promyelocytes^29^. To verify the MPO antibody using for its suitability in Western blot, we used extracts from the HL-60 cell line, a promyelocytic cell line derived from human leukemia^30^, and acute promyelocytic leukemia NB4^31^ as positive controls (Fig. 3D). Under basic conditions, these cell lines lacked LCN2, while incubation with lipopolysaccharides (LPS) or LPS and Tunicamycin (TM) induced expression of LCN2, because LCN2 is produced in later stages of neutrophil maturation and stored in specific secondary granules. Western blot analysis of liver lysates confirmed higher MPO protein levels in wild types compared to the *Lcn2* null mice. In this analysis liver extracts isolated from LPS-injected mice containing high numbers of activated neutrophils directing LCN2 expression were used as positive controls (Fig. 3D, E).

### *Lcn2* null decreased CD45^+^ and CD11b^+^ F4/80^+^ cells in livers after chronic mineral oil administration but not after chronic CCl_4_ challenge

**Fig. 4:**
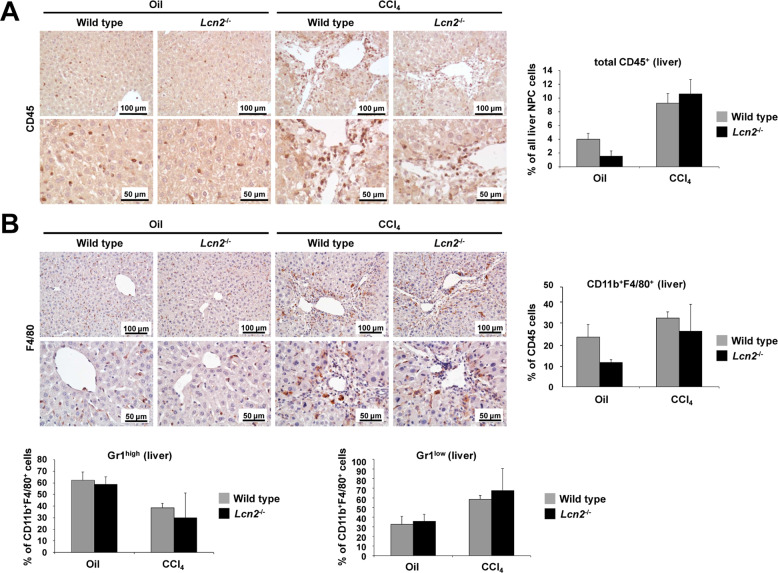
*Lcn2* null decreased CD45^+^ and CD11b^+^ F4/80^+^ cells in livers of chronic mineral oil. **A** IHC staining of CD45^+^ and hepatic leukocyte FACS shows significant increases in mice treated with CCl_4_, but only a marked reduction in *Lcn2*^−/−^ mice receiving oil compared to the wild type and no difference between CCl_4_ treated groups. **B** IHC staining of F4/80 in step with FACS analysis found lesser amounts of CD11b^+^ F4/80^+^ in *Lcn2*^−/−^ mice receiving oil compared to the wild type with no differentiation between CCl_4_ wild type mice and *Lcn2*^−/−^. Mineral oil administration showed high proportions of Gr1^high^ cells, while CCl_4_ treated mice showed high proportions of Gr1^low^ cells.

We next analyzed the panel of other inflammatory infiltrating cells in livers of wild type and *Lcn2* null animals through IHC and FACS and found significantly decreased amounts of CD45^+^ leukocytes, CD11b^+^ F4/80^+^ monocytes, and macrophages in chronic mineral oil administered *Lcn2* null livers compared to wild type, but no difference between the two CCl_4_ groups (Fig. 4A, B). Interestingly, monocytes and macrophages in oil-injected livers showed higher amounts of the Gr1^high^ monocytes, indicating inflammatory responses. By contrast, the CCl_4_ livers showed high proportions of Gr1^low^ monocytes, pointing at chronic wound healing responses, resulting in liver fibrosis.

### *Lcn2* null decreased inflammatory cytokines and chemokines production in repeated mineral oil injection

Since macrophages are the primary producers of cytokines and chemokines, the cytokine and chemokine mRNA levels correlated well with the numbers of monocytes and macrophages. Only oil-injected *Lcn2* null livers showed lesser amounts compared to wild type, while no differences were observed between CCl_4_ wild type and *Lcn*2 null (Supplementary Fig. 1). The M1 macrophage marker *Nos2* was significantly higher in both CCl_4_-treated groups, while the M2 polarization marker *Arg1* was similar in all groups (Supplementary Fig. 1). These findings indicate that *Lcn2* null does affect the inflammatory responses, but not directly in wound healing and fibrogenesis.

### Chronic mineral oil application in wild type mice induced inflammatory leukocyte markers

To confirm that mineral oil application induced liver inflammation, we compared chronic mineral oil**-**injected mice with sham operation and found similar amounts of hepatic *Cd45* mRNA, the marker of total leukocytes, but the mRNA levels such as *Cd11b, F4/80, Ly6G, Mpo* and *Lcn2*, indicators of inflammatory responses, were significantly higher in the wild type receiving mineral oil (Supplementary Fig. 2A). IHC staining confirmed increased numbers of F4/80, MPO, and LCN2 positive cells in mineral oil-injected wild type livers (Supplementary Fig. 2B). In addition, the mRNA of inflammatory cytokines, chemokines, and their receptors such as *Il1a, Il1b, Il1r1, Ccl2, Tnfa*, and *Tnfr1* were significantly upregulated in wild type animals receiving eight-week oil injection. The *Mmp9* gelatinase produced in neutrophils and *Timp1* expression were both increased in wild type oil administered mice, while *Mmp2* were comparable (Supplementary Fig. 2C).

### *Lcn2* null slightly diminished collagen accumulation in chronic CCl_4_-induced fibrosis

**Fig. 5:**
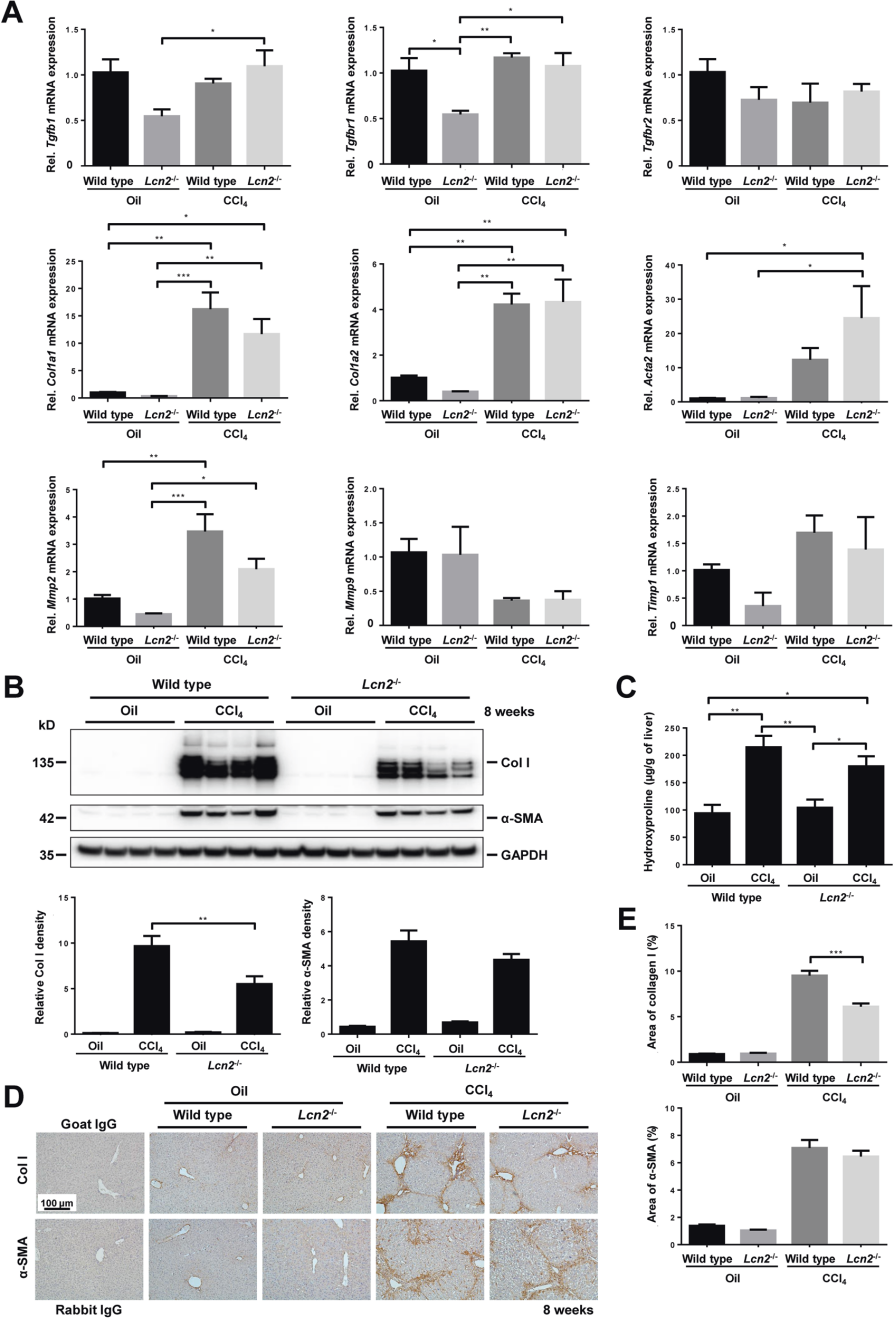
*Lcn2* null decreased collagen accumulation in chronic CCl_4_ induced fibrosis. **A** Quantitative RT-PCR of fibrogenic genes such as *Tgfb1, Tgfbr1, Tgfbr2, Col1a1, Col1a2, Acta2, Mmp2, MMp9*, and *Timp1* only showed significant differences between oil and CCl_4_ treatment, but no difference between wild type and *Lcn2* null animals. **B** Western blot of liver lysates shows significantly higher amounts of collagen type 1 in CCl_4_ treated wild type mice as compared to the *Lcn2*^−/−^ and no detection in oil application groups. No significant difference of α-SMA between wild type and *Lcn2*^−/−^ mice. GAPDH was used as a loading control. The relative Western blot density is shown below. **C** Hydroxyproline assays show significant levels between different groups, but only slightly lower in *Lcn2*^−/−^ mice receiving CCl_4_ compared to the wild type. **D** IHC staining of collagen type 1 and α-SMA shows slightly less collagen deposition in *Lcn2*^−/−^ liver treated with CCl_4_ compared to the wild type and very minimal collagen in oil application groups. Also, a significant increase of α-SMA in CCl_4_ treated livers, but no difference between wild type and *Lcn2*^−/−^ mice. Oil application groups showed only very minimal α-SMA expression. **E** Areas of collagen type 1 and α-SMA deposition were quantified, and only collagen type 1 was significantly higher in the wild type treated CCl_4_ compared to the *Lcn2*^−/−^ and no difference of α-SMA between wild type and *Lcn2*^−/−^ mice.

**Fig. 6:**
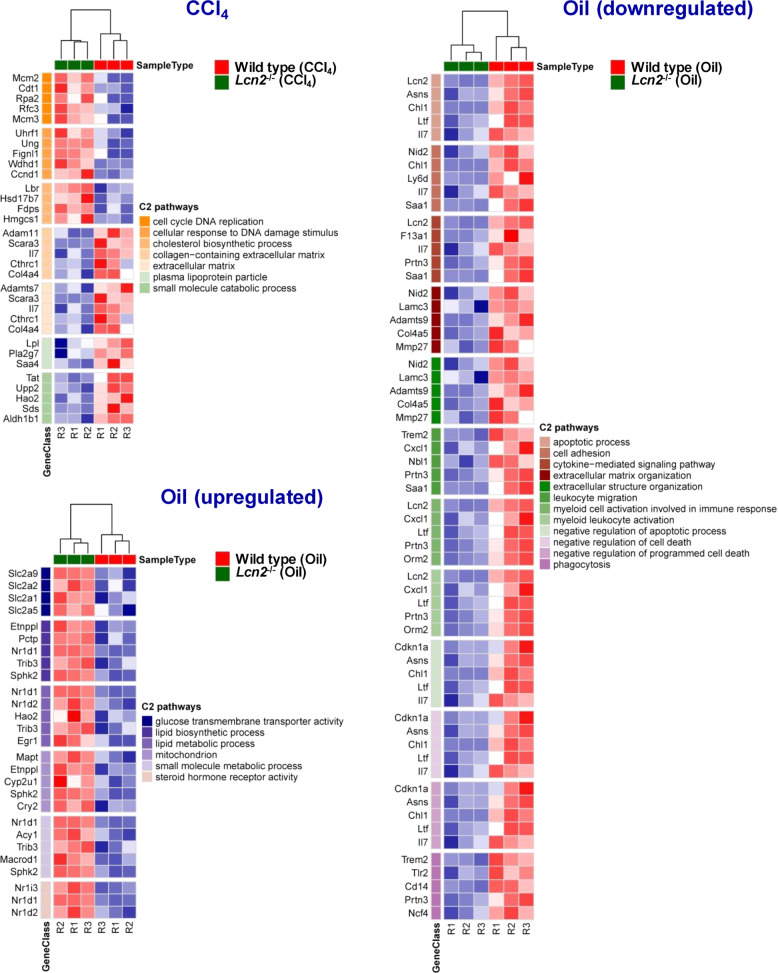
GO heatmaps. *Lcn2* null and wild type mice were compared in chronic CCl_4_ or mineral oil administered groups. The top five genes per gene set were adjusted to the *p* values ≤ 0.05 and gene sorting by Log2 of fold change. Upregulation and downregulation of the gene sets and the compatible pathways as shown.

Although the hepatic leukocyte population assessed by FACS showed no significant difference of liver inflammatory cells between wild type and *Lcn2*^−/−^ in CCl_4_-treated groups (Fig. 4), *Lcn2* null mice showed lower numbers of MPO positive cells and lower levels of MPO protein (Fig. 3). With regard to liver fibrosis, we found no difference in *Tgfb1*, *Tgfbr1*, *Tgfbr2*, *Mmp2*, *Mmp9*, *Timp1*, *Col1a1,* and *Acta2* (α-Sma) mRNA between wild type and *Lcn2*^−/−^ mice receiving CCl_4_ (Fig. 5A), but wild type mice developed slightly more liver fibrosis than *Lcn2*^−/−^ mice as evidenced by higher levels of collagen type I protein in Western blot (Fig. 5B). Measurement of hydroxyproline, a major component of the collagen protein and collagen type I IHC duly confirmed higher levels of fibrosis in wild type mice compared to the *Lcn2*^−/−^. No significant difference was observed in α-SMA mRNA and protein levels and no liver fibrosis in mineral oil administrations (Fig. 5C–E). RNA-Seq confirmed downregulation of collagen-containing extracellular matrix in CCl_4_ administered *Lcn2* null compared to the wild type mice (Fig. 6, Supplementary Figs. 3–5).

### Chronic mineral oil administration suppressed gene sets involved in myeloid leukocyte activation and immune responses in *Lcn2* null mice

Granulocytes and monocytes/macrophages are myeloid lineage cells and are mainly responsible for innate immune responses. We found reduced amounts of liver MPO in IHC staining and Western blot analysis in *Lcn2* null compared to the wild type. Additionally, characterization of liver leukocytes by FACS also showed significantly lower amounts of CD11b^+^ F4/80^+^ monocytes/macrophages in chronic mineral oil administered *Lcn2* null mice compared to the wild type. RNA-Seq results supported our findings that *Lcn2* null livers decreased expression of the gene sets involved in myeloid leukocyte activation, especially neutrophil and monocytes/macrophage functions such as leukocyte migration and cell adhesion as shown in the heat maps (Fig. 6, Supplementary Figs. 3–5).

### *Lcn2* null mice increased lipid biosynthesis

Both mineral oil and CCl_4_ administration did enrich genes involved in lipid biosynthetic and metabolic process such as cholesterol biosynthesis in chronic CCl_4_ application. We found more lipid droplets in both mineral oil and CCl_4_ application in *Lcn2* null compared to wild type mice (data not shown).

### Chronic application of CCl_4_ induced liver cell proliferation in *Lcn2* null more than in wild type mice

**Fig. 7:**
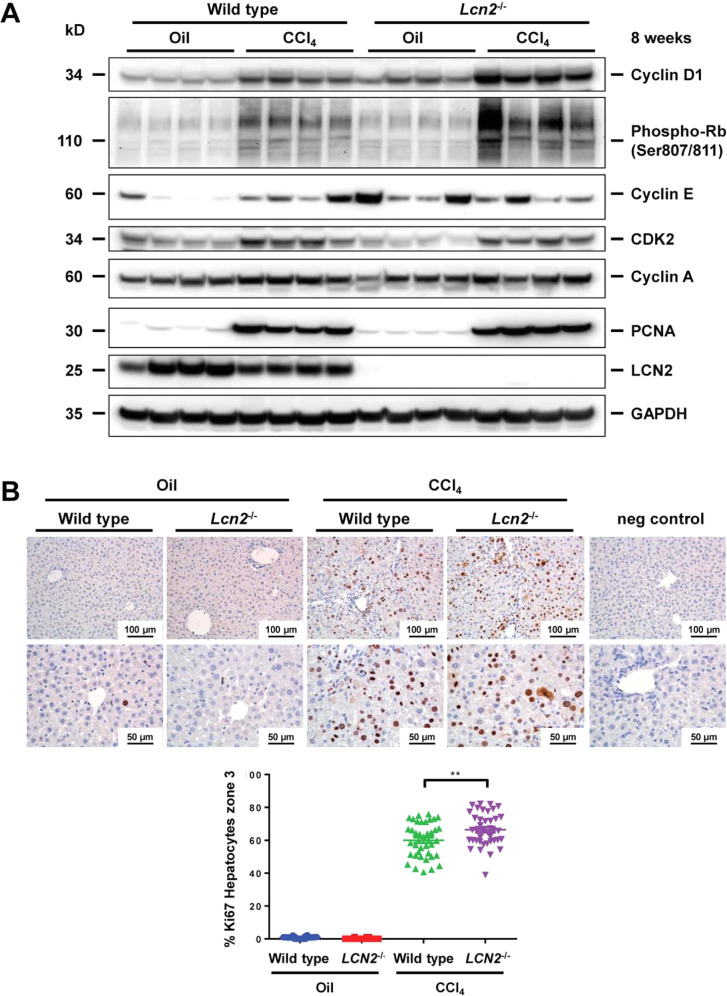
Chronic CCl_4_ application induced liver cell proliferation. **A** Western blots of proteins involved in cell cycle and cell proliferation markers such as cyclin D1, phosphorylated-retinoblastoma (Rb), cyclin E, CDK2, cyclin A, PCNA, and LCN2 as depicted. GAPDH was used as a loading control, and the quantification of Western blots is shown in Supplementary Fig. 6. **B** IHC showed a significant increase in Ki67 positive hepatocyte nuclei in liver zone 3 of the *Lcn2* null compared to the wild type mice.

Surprisingly, RNA-Seq showed significantly enriched gene sets involved in DNA replication and cell cycle in *Lcn2*^*−/−*^ mice receiving eight week CCl_4_ compared to wild type controls (Fig. 6, Supplementary Figs. 3–5). These findings were further confirmed by increased cyclin D1, phosphorylated retinoblastoma protein (Rb), and proliferating cell nuclear antigen (PCNA) in Western blots, while showing no significant differences in cyclin E, CDK2, and cyclin A (Fig. 7A and Supplementary Fig. 6). The antigen identified by monoclonal antibody Ki67 in IHC staining showed significantly increased numbers of Ki67 positive hepatocytes in zone 3 livers of *Lcn2* null compared to the wild type mice (Fig. 7B).

## Discussion

Upon eight week-mineral oil and CCl_4_ application, we unexpectedly found upregulation of *Lcn2* expression in both groups of wild type animals, but no expression in *Lcn2* null mice. Levels of *Lcn2* mRNA and LCN2 protein were significantly higher in mineral oil administered livers. LCN2 IHC staining identified strongly positive NPC, while LCN2 positive staining in CCl_4_ liver was detected both in NPC and hepatocytes around the central veins. LCN2 positive NPC of mineral oil animals was scattered homogeneously throughout the liver but the numbers of cells were significantly lower than in the CCl_4_ treated group. These discrepancies most likely indicate that other cell types such as Kupffer cells and hepatocytes produced LCN2 as well. Especially hepatocytes are the major cell type that produced serum LCN2 in the models of bacterial infection or partial hepatectomy^11^.

Mouse neutrophils are commonly identified based on the cell surface expression of Ly6G and CD11b with the cytoplasmic marker MPO. We performed double staining of LCN2 with Ly6G and LCN2 with MPO and identified the LCN2 positive NPC in oil-treated livers as neutrophils. Neutrophil LCN2 is produced during band cells and segmented cells development and is known as a secondary granule protein^32–34^. Therefore, IHC staining showed LCN2 positive very strongly in neutrophils as compared to the LCN2 in hepatocytes and other cell types such as macrophages, which secrete LCN2 by classical pathways^35^. This explains why the levels of serum LCN2 are significantly higher in wild type animals receiving mineral oil administration than in the fibrotic CCl_4_ group. Intraperitoneal injection of mineral oil leads to the formation of lipo-granulomas of peritoneum and mesenteric lymph nodes consisting of phagocytic cells engulfed the oil droplets^36^. Kupffer cells, the residential macrophages, are the majority of these phagocytic cells in the liver. Upon macrophage activation, they produce cytokines and chemokines attracting neutrophil infiltration as shown in our chronic 8 week-oil administration experiment. These findings are in line with a report from Lee et al. demonstrating that exposure to hydrocarbon oils is associated with the development of chronic inflammation and modulates neutrophil recruitment in peritoneal fluid through IL-1α and IL-1R signaling in a CXCR2-dependent manner^37^. We also found significant mRNA upregulation of several inflammatory cytokines and chemokines including *Il1α*, *Il1β*, *Tnf-α*, *Il6*, *Ccl2*, *Cxcl1*, *Cxcl2*, *Cxcl5* and their receptor mRNAs in chronic mineral oil application of wild type mice.

The inflammatory responses to chronic CCl_4_ intoxication using the mineral oil vehicle as a control, showed mineral oil alone to induce more hepatic inflammation than expected and more severe in wild type than *Lcn2*^−/−^ mice. We found no significant differences between wild type and *Lcn2*^−/−^ receiving CCl_4_ groups, except for the amounts of MPO^+^ cell counts via IHC and Western blot analysis. These findings indicate that combinations of hepatocyte injuries, chronic oxidative stress from CCl_4,_ together with hepatocyte- necrosis, apoptosis, proliferation, and tissue repair lead to liver fibrosis. This process may obscure the differences in inflammatory responses between wild type and *Lcn2*^−/−^ mice. MPO is expressed by circulating neutrophils and monocytes/macrophages in the human liver^38^. We found a 56-kD protein, the predicted molecular mass of MPO (heavy chain) in liver extracts to be comparable between mineral oil- and CCl_4_-injected wild type with similar lower values in *Lcn2*^−/−^ liver after both oil and CCl_4_ administration. Immunofluorescence double staining of liver slides demonstrated co-localization of MPO with LCN2 and LCN2 with Ly6G in mineral oil application neutrophils, while the MPO^+^ cells found along the fibrotic septa in CCl_4_ livers were not all co-localized with LCN2 and Ly6G, but with F4/80 instead. By contrast, we found no co-localization of LCN2 with F4/80, but instead MPO^+^ cells after chronic mineral oil application in both spindle shape (macrophages) and round segmental polymorphonuclear cells located in the sinusoids (Supplementary Fig. 2B). MPO is synthesized during promyelocytes and promyelomonocytes in the bone marrow and terminated as soon as myeloid progenitors enter the myelocyte stage of differentiation^29^. Mature monocytes contain a small size peroxidase in primary granules^39^. The co-localization of MPO and F4/80 cells along the fibrotic central veins is most likely lipid-laden foam cell macrophages engulfed the MPO oxidized low-density lipoprotein (LDL) from CCl_4_ intoxication, which is similar in macrophages at human atherosclerotic lesions^40^.

For CCl_4_-induced liver fibrosis, we found only the significantly increased fibrogenic mRNA gene expression in CCl_4_ treated mice compared to oil administration. Surprisingly, the collagen protein deposition was significantly higher in CCl_4_ treated wild type mice compared to the *Lcn2*^−/−^, as evidenced by Western blot analysis, larger areas stained positive for collagen type 1, and higher levels of hydroxyproline in liver tissue. These findings seem to correspond well with the levels of MPO protein in Western blot and IHC. In line, RNA-Seq results confirmed that the *Lcn2*^*−/−*^ mice provoke defects in gene sets involved in myeloid leukocyte function, especially neutrophil activation during immune responses including leukocyte migration, cell adhesion and neutrophil degranulation. Additionally, *Lcn2*^*−/−*^ mice decreased the expression of genes involved in cell surface receptor signaling, cellular response to cytokine stimulus and cytokine-mediated signaling pathways. For this reason, we found high levels of inflammatory cytokines and chemokines in wild type mice receiving oil compared to the *Lcn2*^*−/−*^ mice (Supplementary Fig. 1).

The effects of LCN2 and liver cell proliferation are still controversial. One particular example of a partial hepatectomy model of both hepatocyte-specific knockout (i.e. *Lcn2*^*Hep–/–*^*)* and *Lcn2*^*−/−*^ mice showed lower rates of liver regeneration, compared to the wild type^11^, while others found that LCN2 does not to alter hepatocyte regeneration^41^. It was the first time in our chronic CCl_4_ model that we found distinct upregulation of gene sets involved in DNA replication and cell cycle in livers of *Lcn2*^*–/–*^ mice receiving CCl_4_ compared to the wild type counterparts (Fig. 6). The cell cycle-regulated proteins such as cyclin D1 and phosphorylated retinoblastoma were significantly higher in *Lcn2*^*−/−*^ livers. In our recent report, we found *Lcn2*^*−/−*^ mice to develop more hepatocyte apoptosis after chronic CCl_4_ application compared to the wild type^21^. This hepatocyte damage may enhance compensating hepatocyte proliferation in *Lcn2*^*−/−*^ mice, as indicated by increased PCNA expression in Western blot and the numbers of Ki67-positive cells in liver zone 3, the most severe hepatocyte injured areas (Figs. 7A and B). The ability of hepatocyte regeneration combined with lower leukocyte inflammatory responses might promote tissue repair and attenuate liver fibrosis in *Lcn2*^*–/–*^ mice. Furthermore, *Lcn2*^−/−^ mice also downregulated gene sets for collagen-containing extracellular matrix structure organization in both oil and CCl_4_ application compared to the wild type counterparts.

In summary, chronic mineral oil intraperitoneal administration induced hepatic inflammation through innate immune responses via myeloid cell activation. The *Lcn2* null mice developed less inflammation due to defects in genes involved in the myeloid cell activation process, and downregulated gene sets for collagen-containing extracellular matrix leading to mitigation of the liver fibrosis. Additionally, it is the first time that we found *Lcn2* null mice to show enrichment of genes responsible for DNA damage and cell cycle DNA replication, compared to the wild type mice upon chronic CCl_4_ liver injury.

## Supplementary information

Supplemental Material

## Data Availability

The datasets used and/or analyzed during the current study are available from the corresponding author on reasonable request. Expression data is accessible via the Gene Expression Omnibus (GEO) under accession number GSE176093.
