# Supplementary material for: Chronic mineral oil administration increases hepatic inflammation in wild type mice compared to lipocalin 2 null mice
Source: Lab Invest. 2021 Sep 13;101(12):1528–39. doi: 10.1038/s41374-021-00672-9 (PMC8590977; doi:10.1038/s41374-021-00672-9)
Supplement: Supplementary file 1 — Supplemental Material [file 41374_2021_672_MOESM1_ESM.pdf]

Supplemental Material

Chronic mineral oil administration increased hepatic inflammation in wild type mice compared to Lipocalin 2 null mice

Erawan Borkham-Kamphorst[1], Ute Haas[1], Manuela Pinoé-Schmidt[1], Ali T. Abdallah[2] and Ralf Weiskirchen[1]

[1] Institute of Molecular Pathobiochemistry, Experimental Gene Therapy and Clinical Chemistry, RWTH Aachen University Hospital, Aachen, Germany

[2] Interdisciplinary Center for Clinical Research, University Hospital RWTH, Aachen, Germany

Supplementary Tables

Suppl. Table 1: Primers used in this study.

Suppl. Table 2: Antibodies used in this study.

Supplementary Figures

Suppl. Figure 1: Cytokine and chemokine expression after administration of mineral oil or carbon tetrachloride.

Suppl. Figure 2: Wild type mice receiving mineral oil developed more liver inflammation compared to sham.

Suppl. Figure 3: Similarity heatmaps with hierarchical clustering for all four comparisons.

Suppl. Figure 4: MA plots for each of the comparisons.

Suppl. Figure 5: Heatmaps of top enriched GO terms.

Suppl. Figure 6: Western blot quantification.

Suppl. Table 1: Primers used in this study

Gene	Acc. No.	Primer
Lcn2	NM_008491	for: 5’-CCATCTATGAGCTACAAGAGAACAAT-3’<br>rev: 5’-TCTGATCCAGTAGCGACAGC-3’
Cd45	NM_001111316	for: 5’-CTGACAATCCCACACTCACG-3’<br>rev: 5’-TCCCCGGTACAGTCCTCTC-3’
Cd11b	NM_001082960	for: 5’-CAATAGCCAGCCTCAGTGC-3’<br>rev: 5’-GAGCCCAGGGGAGAAGTG-3’
F4/80	NM_010130	for: 5’-GGAGGACTTCTCCAAGCCTATT-3‘<br>rev: 5’-AGGCCTCTCAGACTTCTGCTT-3’
Ly6G	NM_001310438	for: 5’-TTGTGGTCCTACTGTGTGCAG-3’<br>rev: 5’-TCAGGTGGGACCCCAATAC-3’
Mpo	NM_010824	for: 5’-GATGGAATGGGGAGAAGCTC-3’<br>rev: 5’-GCAGGTAGTCCCGGTATGTG-3’
Ccl2	NM_011333	for: 5’-GTGTTGGCTCAGCCAGATGC-3’<br>rev: 5’-GACACCTGCTGCTGGTGATCC-3’
Ccr2	NM_009915	for: 5’-ACCTGTAAATGCCATGCAAGT-3’<br>rev: 5’-TGTCTTCCATTTCCTTTGATTTG-3’
Cxcl1	NM_008176	for: 5’-AGACTCCAGCCACACTCCAA-3’<br>rev: 5’-TGACAGCGCAGCTCATTG-3’
Cxcl2	NM_009140	for: 5’-AAAATCATCCAAAAGATACTGAACAA-3’<br>rev: 5’-CTTTGGTTCTTCCGTTGAGG-3’
Cxcl5	NM_009141	for: 5’-TAGAGCCCCAATCTCCACAC-3’<br>rev: 5’-GGAGCTGGAGGCTCATTGT-3’
Cxcr2	NM_009909	for: 5’-CAGGACCAGGAATGGGAGTA-3’<br>rev: 5’-TCCCCTCCAAATATCCCCTA-3’
IFNγ	NM_008337	for: 5’-GGAGGAACTGGCAAAAGGATGG-3’<br>rev: 5’-TGTTGCTGATGGCCTGATTGTC-3’
Il1a	NM_010554	for: 5’-TTGGTTAAATGACCTGCAACA-3’<br>rev: 5’-GAGCGCTCACGAACAGTTG-3’
Il1b	NM_008361	for: 5’-GAGCTGAAAGCTCTCCACCTC-3’<br>rev: 5’-CTTTCCTTTGAGGCCCAAGGC-3’
Il1r1	NM_001123382	for: 5’-ATTGTTGAACATCGCCACTG-3’<br>rev: 5’-AAATGAGCCCCAGTAGCACTT-3’
Il10	NM_010548	for: 5’-GGCTGAGGCGCTGTCATCG-3’<br>rev: 5’-CTCTAGACTTCGAGCAGGAGATGG-3’
Tnfa	NM_013693	for: 5’-ACCACGCTCTTCTGTCTACTGA-3’<br>rev: 5’-TCCACTTGGTGGTTTGCTACG-3’
Tnfr1	NM_011609	for: 5’-GGAAAGTATGTCCATTCTAAGAACAA-3’<br>rev: 5’-AGTCACTCACCAAGTAGGTTCCTT-3’
Tgfb1	NM_011577	for: 5’-TGGAGCAACATGTGGAACTC-3’<br>rev: 5’-CAGCAGCCGGTTACCAAG-3’
Tgfbr1	NM_009370	for: 5’-GCAGCTCCTCATCGTGTTG-3’<br>rev: 5’-AGAGGTGGCAGAAACACTGTAAT-3’
Tgfbr2	NM_009371	for: 5’-AGAAGCCGCATGAAGTCTG-3’<br>rev: 5’-GGCAAACCGTCTCCAGAGTA-3’
Nos2	NM_010927	for: 5’-CTTTGCCACGGACGAGAC-3’<br>rev: 5’-TCATTGTACTCTGAGGGCTGAC-3’
Il6	NM_031168	for: 5’-GCTACCAAACTGGATATAATCAGGA-3’<br>rev: 5’-CCAGGTAGCTATGGTACTCCAGAA-3’
Arg1	NM_007482	for: 5’-GAATCTGCATGGGCAACC-3’<br>rev: 5’-GAATCCTGGTACATCTGGGAAC-3’
Col1a1	NM_007742	for: 5’-CATGTTCAGCTTTGTGGACCT-3’<br>rev: 5’-GCAGCTGACTTCAGGGATGT-3’

Col1a2	NM_007743	for: 5'-CAAGCATGTCTGGTTAGGAGAG-3'<br>rev: 5'-AGGACACCCCTTCTACGTTGT-3'
Acta2<br>(*α-Sma*)	NM_009606	for: 5'-AATGAGCGTTTCCGTTGC-3'<br>rev: 5'-ATCCCCGCAGACTCCATAC-3'
Mmp2	NM_008610	for: 5'-AACTTTGAGAAGGATGGCAAGT-3'<br>rev: 5'-TGCCACCCATGGTAAACAA-3'
Mmp9	NM_013599	for: 5'-ACGACATAGACGGCATCCA-3'<br>rev: 5'-GCTGTGGTTCAGTTGTGGTG-3'
Timp1	NM_001044384	for: 5'-GCAAAGAGCTTTCTCAAAGACC-3'<br>rev: 5'-AGGGATAGATAAACAGGGAAACACT-3'
Gapdh	NM_008084	for: 5'-TGTTGAAGTCACAGGAGACAACCT-3'<br>rev: 5'-AACCTGCCAAGTATGATGACATCA-3'

Suppl. Table 2 :Antibodies used in this study

Antibody	Cat. No.	Supplier	Host / Clonality	Species	Dilution*
LCN2	AF1857	R&D Systems, Wiesbaden, Germany	Goat / poly	m, r	1:1,000 (WB) 1:50 (IHC)
Ly6G	ab210204	Abcam, Berlin, Germany	Rat / mono	m	1:50 (IHC)
CD45	ab10558	Abcam	Rabbit / poly	h, m, r	1:100 (IHC)
MPO	ab9535	Abcam	Rabbit / poly	h, m	1:50 (IHC)
MPO	HP9048	Hycult Biotech, Uden, The Netherlands	Rabbit / poly	h, m	1:1,000 (WB) 1:50 (IHC)
F4/80	ab2409461	Abcam	Rabbit / poly	m	1:200 (IHC)
F4/80	MCA497R	Serotec, Bio-Rad, Puchheim, Germany	Rat / mono	m	1:1,000 (WB) 1:50 (IHC)
α-SMA	ab32575	Abcam	Rabbit / mono	h, m, r	1:1,000 (WB) 1:200 (IHC)
Collagen type I	1310-01	SouthernBiotech, Biozol, Eching, Germany	Goat / poly	h, m, r	1:1,000 (WB) 1:100 (IHC)
GAPDH	sc-32233	Santa Cruz Biotech., Santa Cruz, CA	Mouse / mono	h, m, r	1:1,000 (WB)
Cyclin D1	2978	Cell Signaling, Darmstadt, Germany	Rabbit / mono	h, m, r	1:1,000 (WB)
p-Rb	9308	Cell Signaling	Rabbit / poly	h, r, mk	1:1,000 (WB)
Cyclin E	07-687	Sigma-Aldrich, Darmstadt, Germany	Rabbit / poly	m, r	1:1,000 (WB)
CDK2	sc-748	Santa Cruz	Rabbit / poly	h, m, r	1:1,000 (WB)
Cyclin A	sc-751	Santa Cruz	Rabbit / poly	h, m, r	1:1,000 (WB)
PCNA	sc-56	Santa Cruz	Mouse / mono	h, m, r	1:1,000 (WB)
Ki67	ab16667	Abcam	Rabbit / mono	h, m, r	1:200 (IHC)
β-actin	A5441	Sigma	Mouse / mono	h, m, r	1:10,000 (WB)

Abbreviations used are: mono, monoclonal antibody; IHC, immunohistochemistry; WB, Western blot; poly, polyclonal antibody; h, human; m, mouse; r, rat, mk, monkey. Monoclonal antibodies used for FACS analysis were obtained from FisherScientific (CD11b) or BD Pharmingen (Gr1/Ly6C).

Supplementary Figures

Suppl. Figure 1: Cytokine and chemokine expression after administration of mineral oil or carbon tetrachloride. Wild type mice increased inflammatory cytokine and chemokine production in repeated mineral oil injection. Quantitative RT-PCR of cytokines and chemokines and their receptors such as *Tnfa, Tnfr1, Il1a, Il1b, Il1r1, Il6, Il10, Ifng, Ccl2, Ccr2, Cxcl1, Cxcl2, Cxcl5, and Cxcr2* remain high in wild type mice receiving mineral oil. M1 macrophage marker *Nos2* was high in CCl_4-treated mice, with no change in M2 macrophage marker *Arg1*.

Suppl. Figure 2: Wild type mice receiving mineral oil developed more liver inflammation compared to sham. (A) Quantitative RT-PCR shows of the leukocyte surface markers such as *Cd45, Cd11b, F4/80, Ly6G, Mpo,* and *Lcn2,* although no difference in *Cd45* the marker of total leukocytes, However, the other inflammatory leukocyte markers were significantly higher in the wild type mice treated with mineral oil for 8 weeks compared to sham including *Lcn2.* **(B)** IHC showed markedly increased hepatic infiltration of F4/80 and MPO positive staining as well as the LCN2 positive non-parenchymal cells in wild type mice receiving 8-week oil compared to sham operation. **(C)** Quantitative RT-qPCR of inflammatory cytokines, chemokines, and their receptors were significantly higher in wild type animals receiving 8 week-mineral oil, such as *Il1a, Il1b, Il1r1, Ccl2, Tnfa, and Tnfr1*. Markedly high levels of *Mmp9* and *Timp-1* were detected in oil administration groups, while no difference in *Mmp2* compared to sham-operated animals.

Suppl. Figure 3: Similarity heatmaps with hierarchical clustering for all four comparisons. The used similarity metric is the euclidean distance. The underlying

expression matrix is the normalized one based on the variance stabilization transformation implemented in the DESeq package. The darkest red means samples are identical; the darkest blue means samples are very dissimilar. Conditions are generally showing the expected clustering and similarities. Only in the first comparison do we notice one replicate from each condition that is not clustering like usually anticipated. But here, we also see that sample similarity within each condition is generally not as high as in the other comparisons, and sample dissimilarity across conditions is not as high as in the other comparisons. That may be due to weaker phenotypic differences, which are sensitive to low technical bias.

Suppl. Figure 4: MA plots for each of the comparisons. An MA plot is a scatter plot of log2 fold changes (on the y-axis) against the average expression signal (on the x-axis). In this regard, we are using the baseMean values from the output of the DESeq package for the mean expression value and the log2 fold changes from the same output also. Each gene with absolute fold change ≥ 1.5 and adjusted *p*-value ≤ 0.05 are considered as significant. Blue dots refer to downregulated genes, red dots to upregulated genes, and grey ones are non-significant genes. We show the top 10 regulated genes. We see *Lcn2* among the top downregulated genes in the first two comparisons, which is a good quality check.

Suppl. Figure 5: Heatmaps of top enriched GO terms. For each comparison, the heatmap of the top variable genes from top enriched GO terms is shown. First, we select the top 10 positively enriched and the top 10 negatively enriched GO terms. From these, we extract all genes significantly regulated as computed by the gprofiler2 (see method description) package. Then, these genes are sorted by variance across all

samples, and the top 50 variable genes are selected. The underlying expression matrix is the normalized one using the variance stabilization transformation of the DESeq2 package. Finally, the hierarchical clustering is performed based on the euclidean distance.

Suppl. Figure 6: Western blot quantification. Western blot quantification of Figure 7A in relation to GAPDH shows significantly increased cyclin D1, and phosphorylated retinoblastoma protein (Rb), and PCNA, while no significant differences in cyclin E, CDK2, and cyclin A. The LCN2 protein increased in wild type livers in both mineral oil and CCl_4 application.

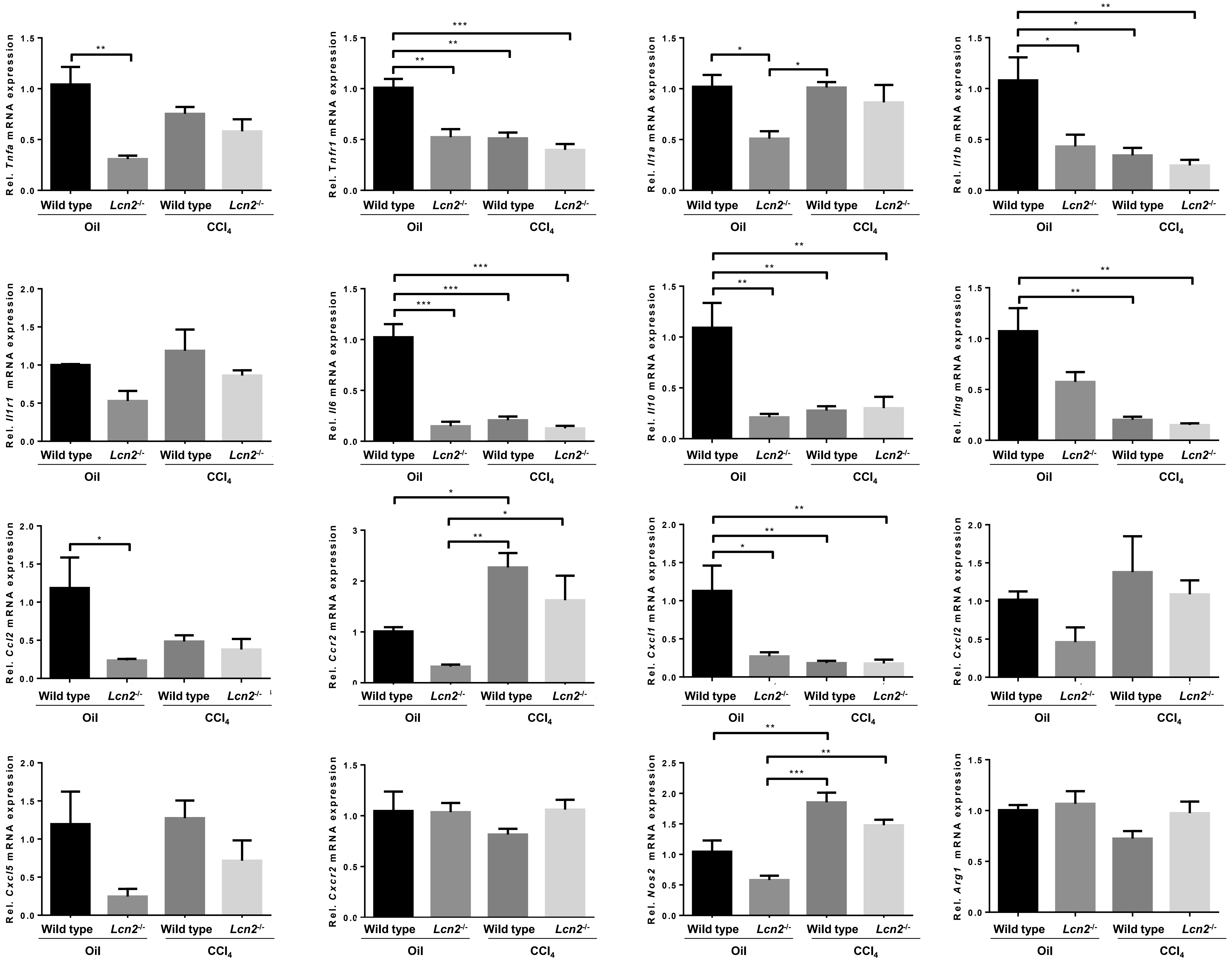

Suppl. Figure 1

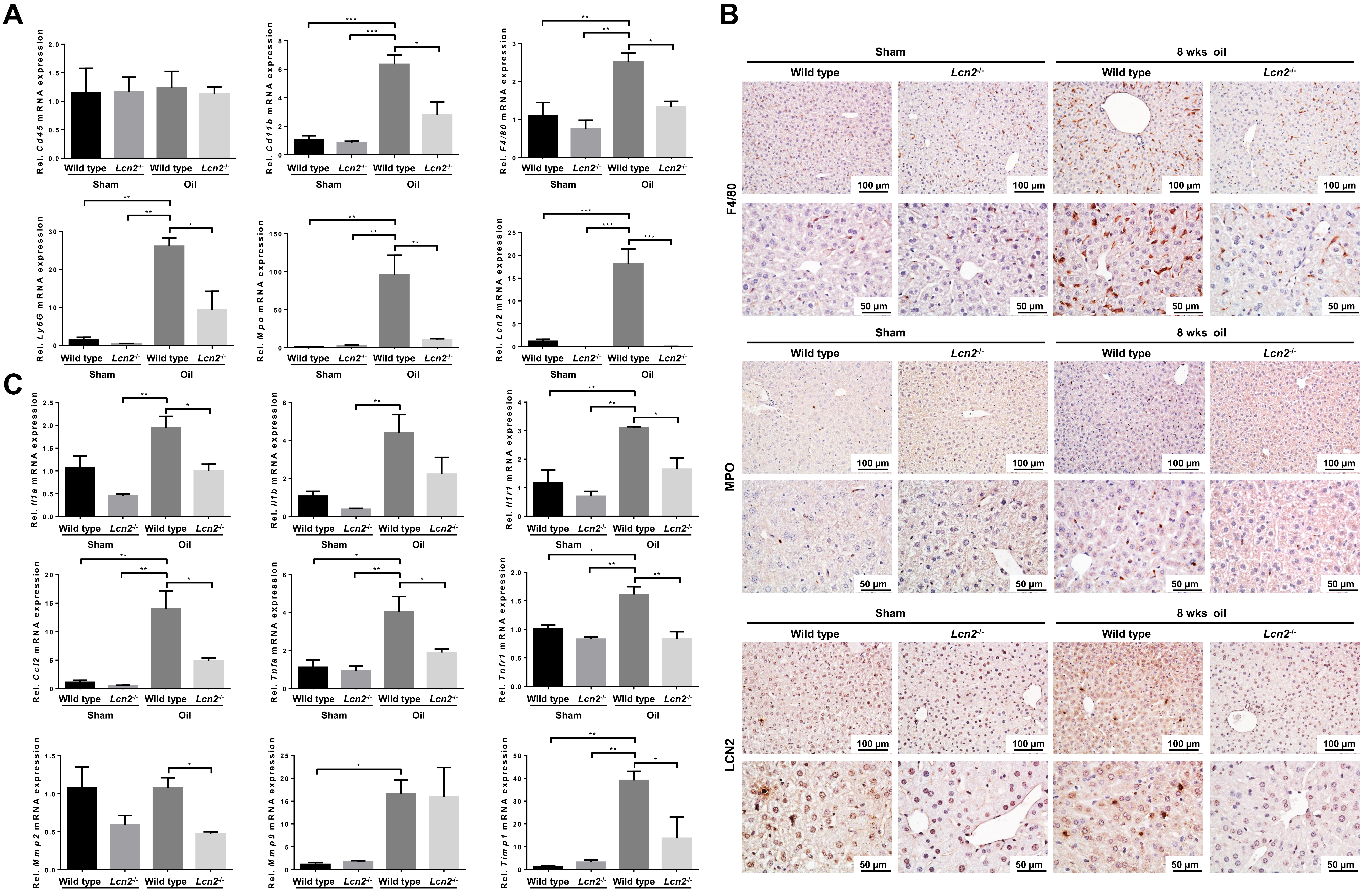

Suppl. Figure 2

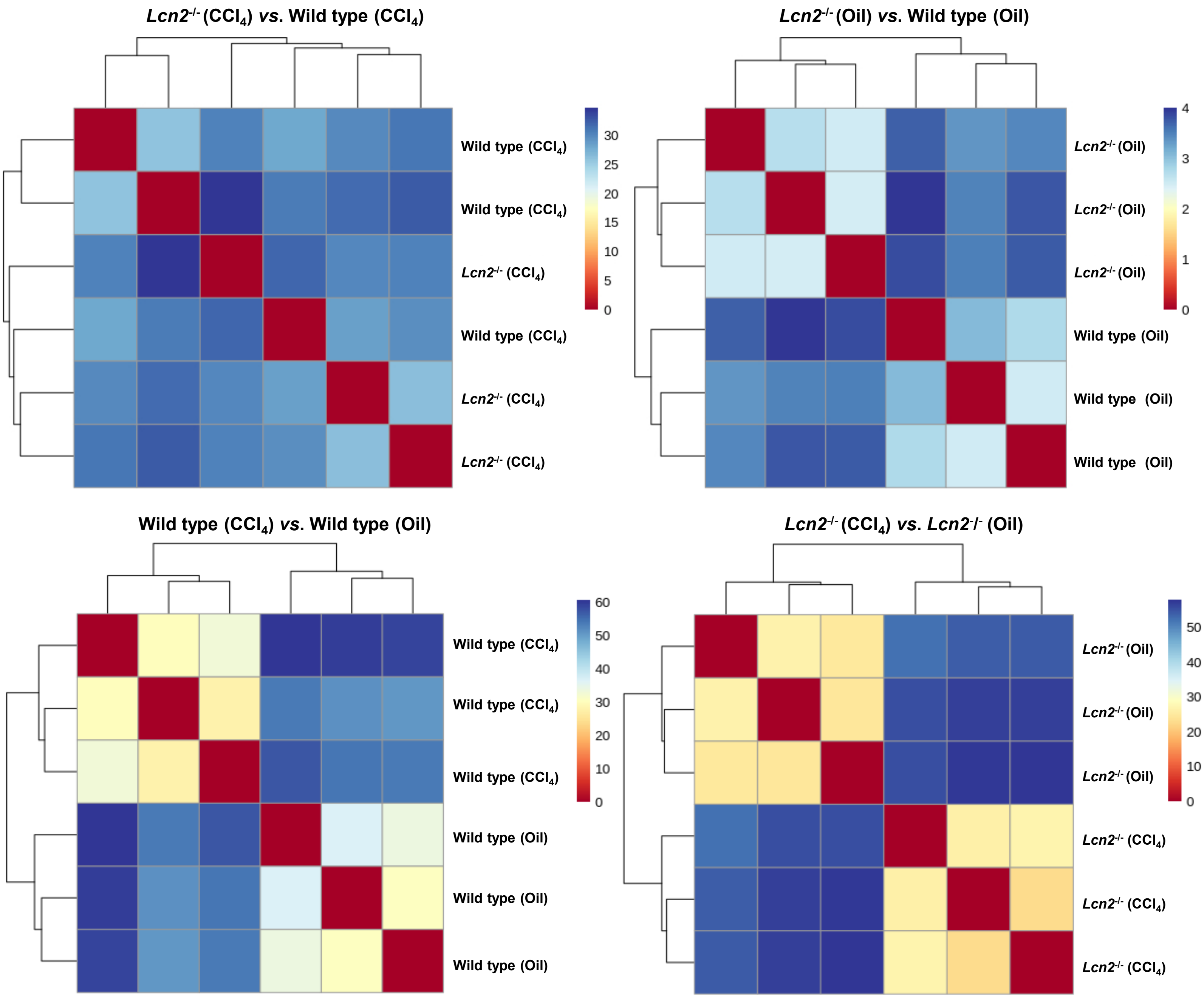

Suppl. Figure 3

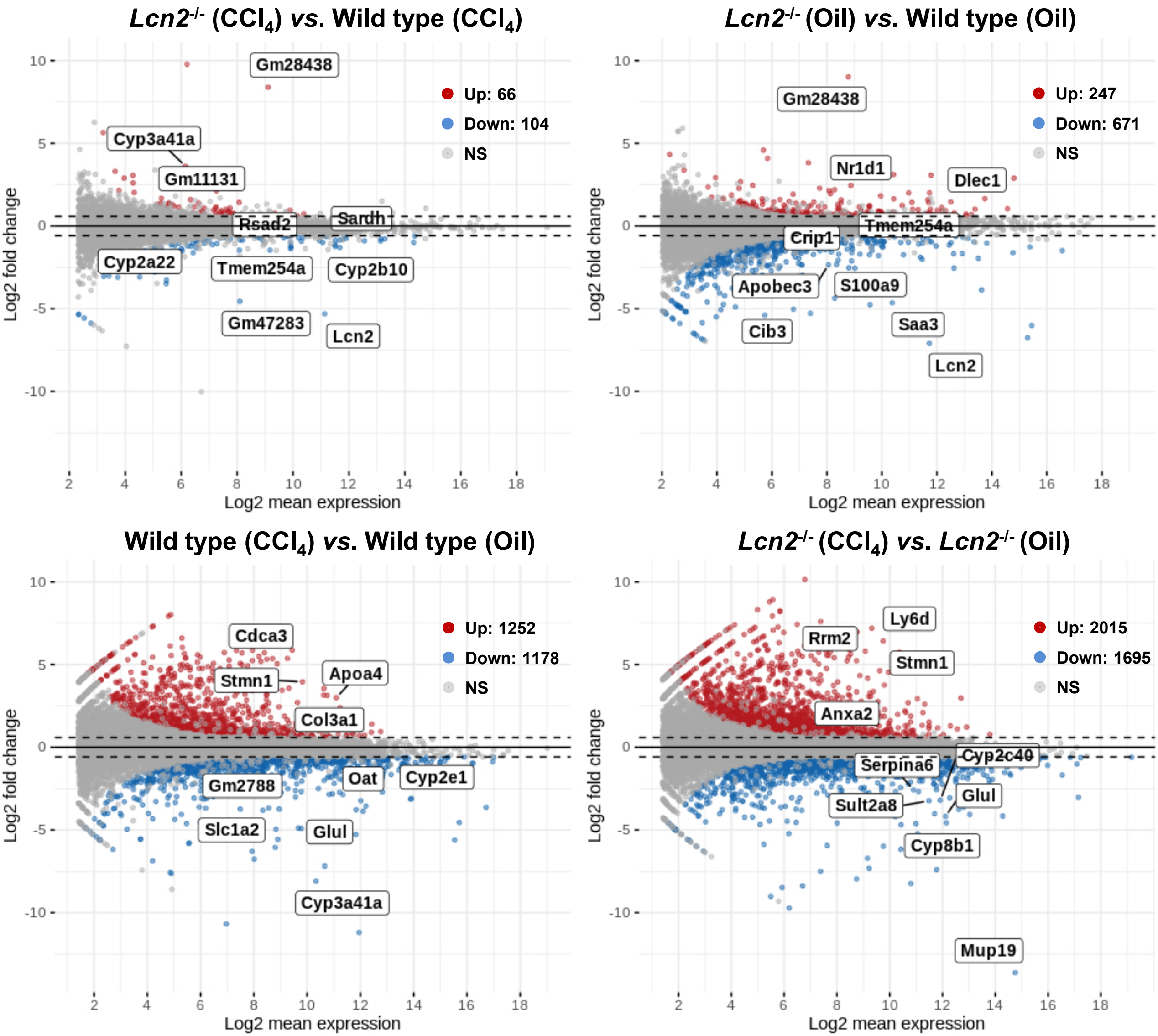

Suppl. Figure 4

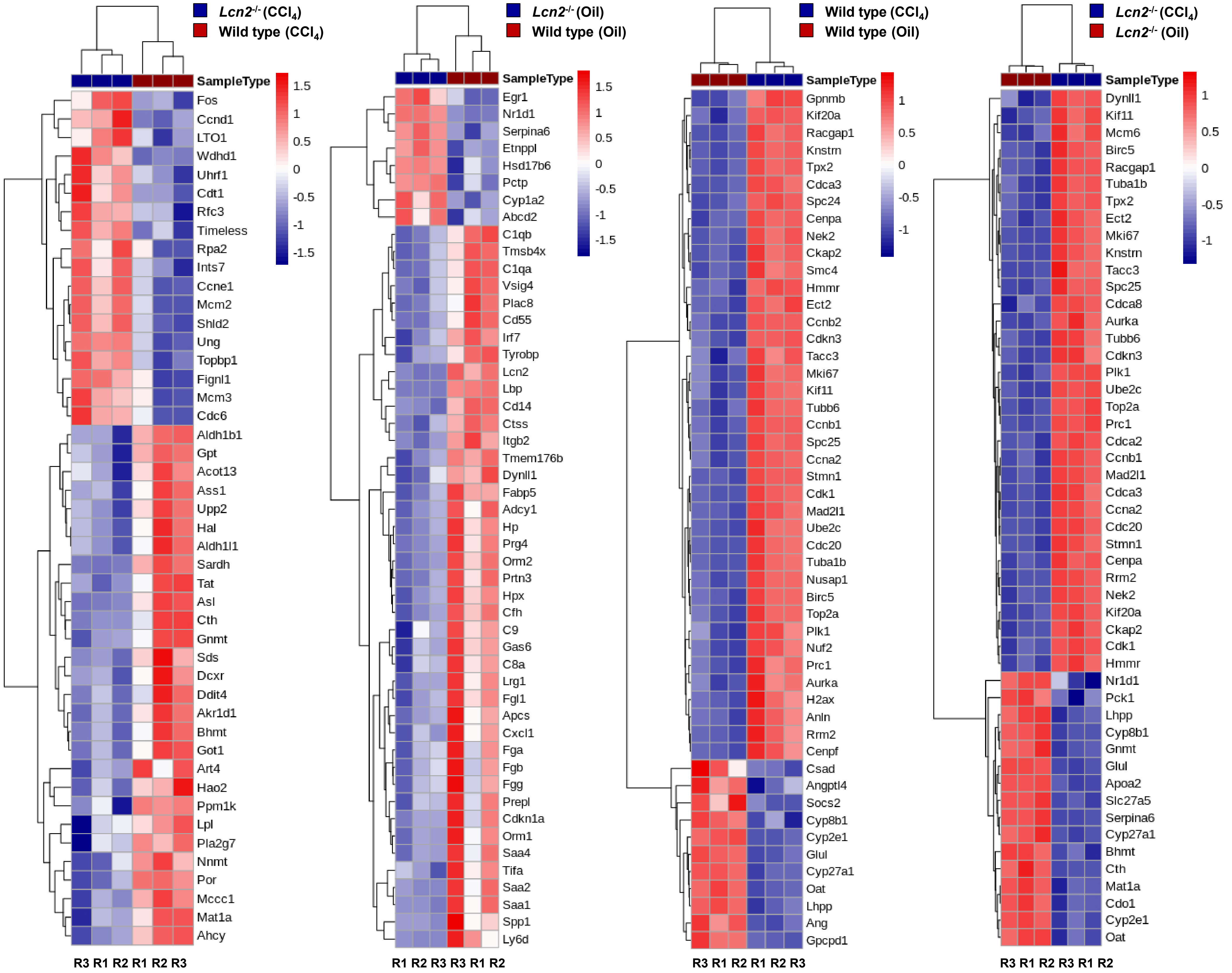

Suppl. Figure 5

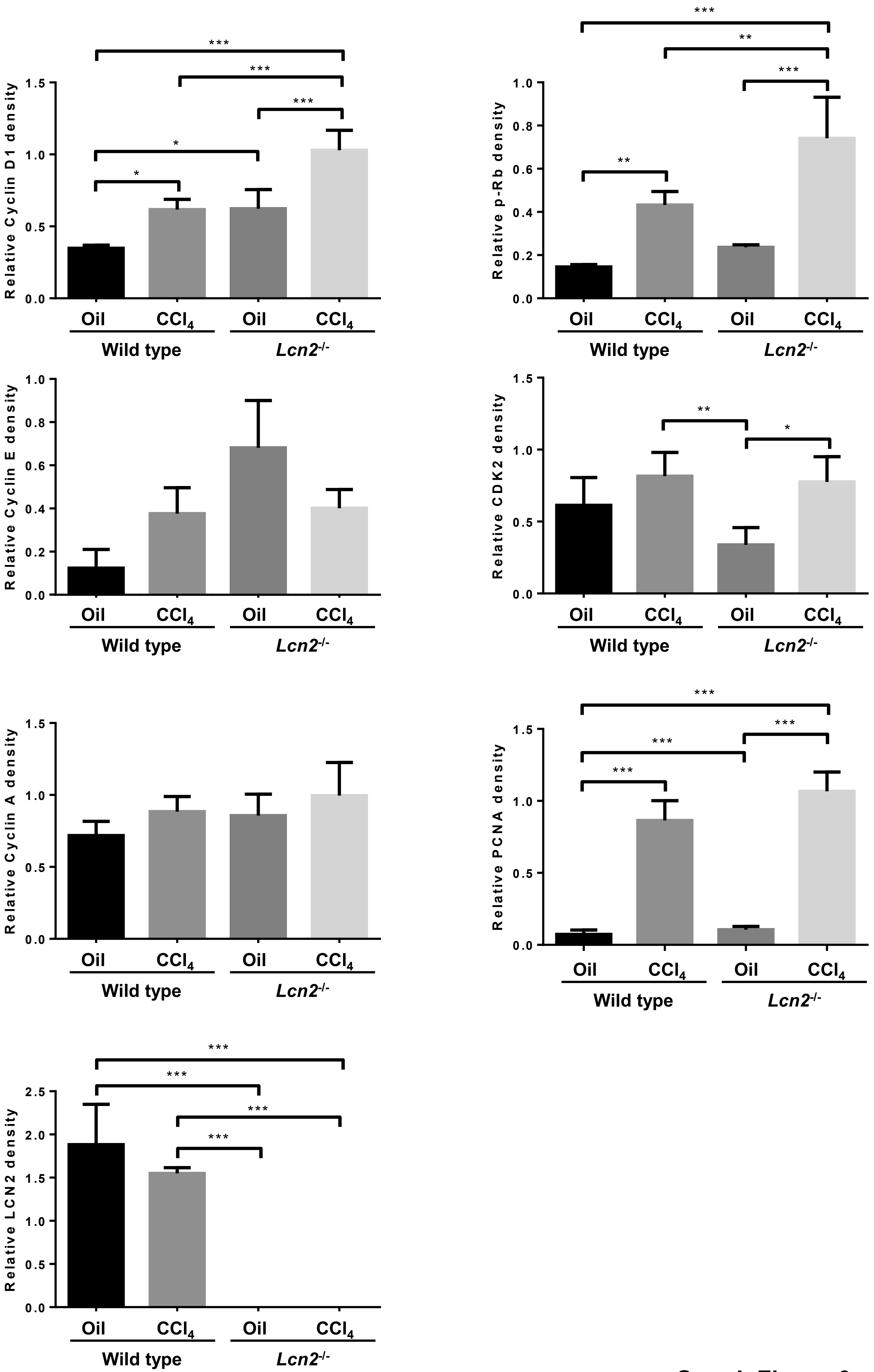

Suppl. Figure 6
